# Auricular Reconstruction Following Traumatic Amputation: A Historical Review and Contemporary Treatment Algorithm

**DOI:** 10.3390/jcm15186942

**Published:** 2026-09-08

**Authors:** Benedikt Fuchs, Vanessa Lucksch, Constanze Kuhlmann, Irene Mesas Aranda, Nikolaus Thierfelder, Sinan Mert, Paul Severin Wiggenhauser

**Affiliations:** Division of Hand, Plastic and Aesthetic Surgery, LMU University Hospital, LMU Munich, 80336 Munich, Germany; vanessa.lucksch@med.uni-muenchen.de (V.L.); constanze.kuhlmann@med.uni-muenchen.de (C.K.); irene.mesas@med.uni-muenchen.de (I.M.A.); nikolaus.thierfelder@med.uni-muenchen.de (N.T.); sinan.mert@med.uni-muenchen.de (S.M.); severin.wiggenhauser@med.uni-muenchen.de (P.S.W.)

**Keywords:** ear amputation, history of ear reconstruction, auricular reattachment methods, auricular reconstruction methods, therapeutic algorithm

## Abstract

**Background**: Traumatic auricular amputation represents a rare but complex reconstructive challenge, and numerous surgical techniques have been developed over the past century to optimize functional and aesthetic outcomes. This historical review examines current auricular reattachment and replantation strategies by comparing established surgical techniques for traumatic auricular amputation. **Methods**: A historical literature search was conducted in PubMed to identify relevant case reports on auricular reconstruction following amputation, dating from 1898 to the present. Eligible studies were reviewed according to the surgical technique used: composite graft reattachment, local flap, pocket techniques, rib cartilage reconstruction, microsurgical replantation, and tissue engineering. Key milestones were documented chronologically and visualized in a timeline. **Results**: A total of six major reconstructive approaches were identified in the literature, highlighting the historical evolution of auricular replantation methods from 1898 to 2018. Compiled data were visualized to underscore the procedural development and categorize surgical innovations over time. **Conclusions**: This review presents a synthesized treatment algorithm that integrates historical context and modern adaptations of auricular replantation techniques. The integrated approach provides a structured framework for managing auricular amputations and promotes standardized practices to optimize functional and aesthetic outcomes in auricular reconstructive surgery.

## 1. Introduction

Auricular injuries are a common clinical entity, particularly in the context of maxillofacial trauma, and may result from a broad spectrum of etiologies. Road traffic accidents represent the leading cause of auricular trauma, followed by bite injuries inflicted by both humans and animals [1]. Among these, human bite injuries account for a substantial proportion of cases and pose unique reconstructive challenges due to tissue loss, contamination, and the complex anatomy of the external ear. Bardsley and Mercer [2] identified facial bite wounds as a frequent form of traumatic injury, while Stucker et al. reported auricular involvement in 25% of patients presenting with facial bite trauma [3].

The clinical and societal relevance of auricular injuries was notably highlighted by the widely publicized incident during a professional boxing match in June 1997, in which Mike Tyson bit off a portion of Evander Holyfield’s auricle [4]. The increasing popularity of body modification practices has contributed to a growing incidence of auricular injuries [5], emphasizing the need for a standardized classification system and evidence-based treatment algorithms to optimize clinical management and reconstructive outcomes [6].

To address the variability and complexity of auricular injuries, we use a modified classification system based on the framework established by Laskin and Donohue in 1958 [7]. This refined system categorizes auricular injuries into four severity grades [8]: Grade I: lacerations with minimal cartilage involvement; Grade II: tear wounds with a nourishing skin bridge; Grade III: avulsions without segment loss, where the detached auricular segment remains available for reattachment, including partial segment detachment and complete auricular avulsion; Grade IV: avulsions with segment loss, where the detached auricular segment is missing.

Particular emphasis is placed on Grade III auricular injuries, as prompt and appropriate intervention may allow successful replantation of the amputated auricular tissue, thereby achieving favorable functional and aesthetic outcomes. Given the considerable diversity of reconstructive techniques described in the literature, a comprehensive understanding of the available treatment options is essential for informed clinical decision-making.

Accordingly, this review examines both established replantation techniques and their historical development, highlighting key milestones that have shaped contemporary approaches to auricular reconstruction. Furthermore, these conventional methods are critically compared with modifications of the pocket technique originally described by Sexton and subsequently refined by Mladick and colleagues [9,10].

The primary aim of this review is to provide a comprehensive overview of current reconstructive strategies while offering practical recommendations for routine clinical practice. By proposing a structured framework for the diagnosis, classification, treatment planning, and prognostic assessment of auricular injuries, this work seeks to facilitate standardized clinical management, improve patient outcomes, and further advance the field of auricular reconstructive surgery.

## 2. Materials and Methods

A narrative literature search was conducted using PubMed (MEDLINE) for publications indexed from 1898 onward. The search strategy combined Medical Subject Headings (MeSH) and free-text terms relating to the external ear, traumatic amputation or avulsion, reattachment or replantation, and reconstructive techniques, including composite grafts, pocket techniques, local flaps, rib cartilage reconstruction, microsurgery, and tissue engineering. The complete PubMed search strategy is provided in the Supplementary Materials.

The search identified 153 records. All records were screened by title and abstract, of which 102 were excluded. The remaining 51 publications underwent full-text assessment. Publications were eligible if they (1) described traumatic auricular amputation or avulsion, (2) reported a reconstructive, reattachment, or replantation procedure, (3) provided sufficient information to identify the reconstructive technique, (4) were published in English, and (5) represented an initial or landmark description of a distinct reconstructive approach. Reports describing non-amputative injuries, congenital or developmental deformities, infections without traumatic amputation, or previously established reconstructive techniques were excluded. Following full-text assessment, three publications met the predefined eligibility criteria.

The six principal reconstructive categories were predefined based on the major reconstructive strategies identified during a preliminary assessment of the literature. Because the primary objective was to describe the historical evolution of auricular reconstruction rather than to assess comparative treatment efficacy, no formal quality or risk-of-bias assessment was performed.

Given that many seminal reports on auricular amputation predated comprehensive electronic indexing, the PubMed search was complemented by manual screening of reference lists of relevant publications and historical surgical literature, including Weerda’s Chirurgie der Ohrmuschel—Verletzungen, Defekte und Anomalien [8]. This additional historical search identified 15 further reports describing distinct reconstructive approaches, resulting in a total of 18 publications included in the historical analysis.

**Figure 1:**
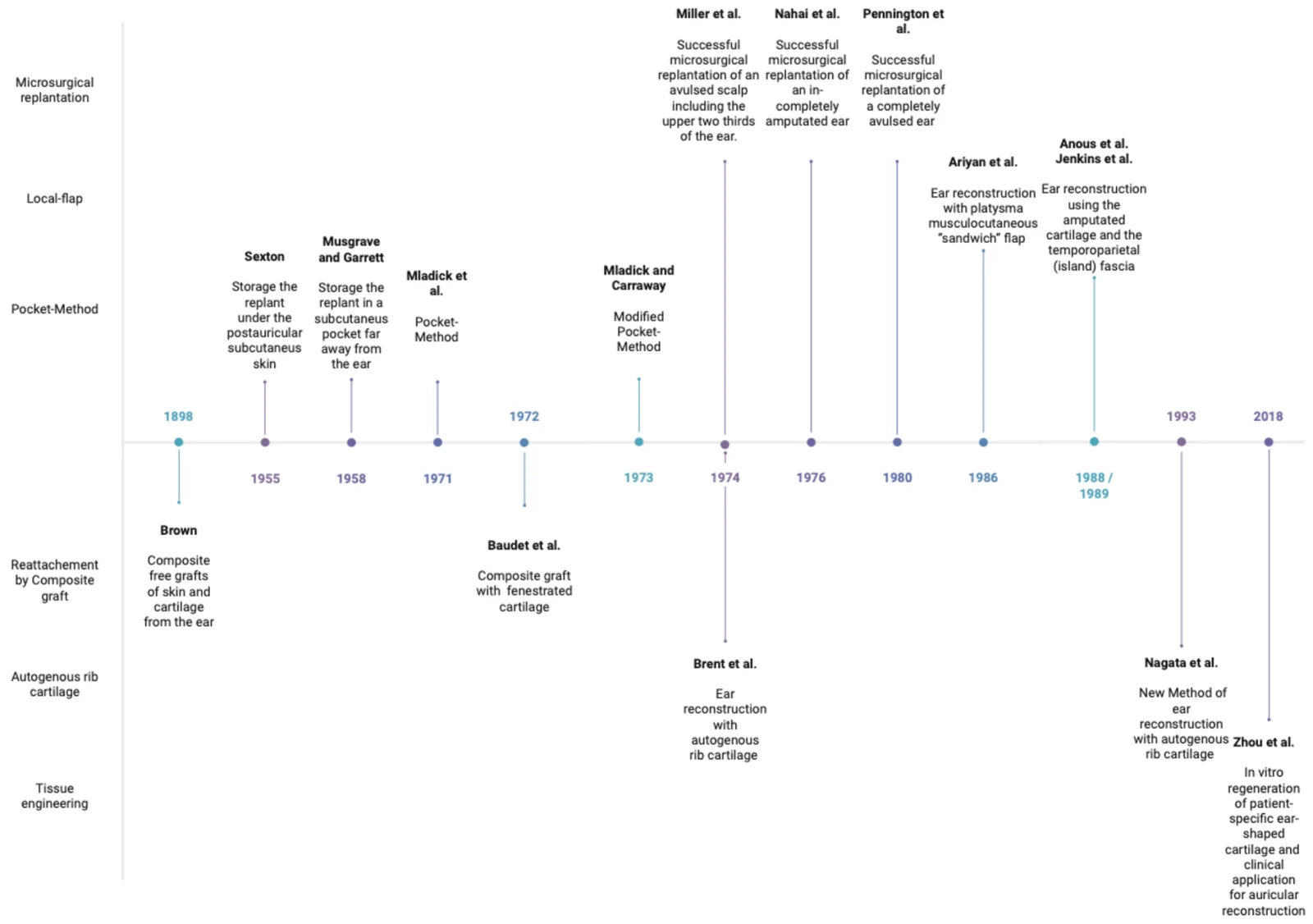
Overview of historical milestones in reconstructive methodologies [9,10,11,12,13,14,15,16,17,18,19,20,21,22,23]. Created in BioRender. https://app.biorender.com/illustrations/.

The publications were categorized according to the predefined reconstructive strategies: (1) composite graft reattachment, (2) pocket techniques, (3) local flap reconstruction, (4) autologous rib cartilage reconstruction, (5) microsurgical replantation, and (6) tissue-engineering approaches. For each publication, the first author, year of publication, reconstructive technique, and relevant clinical and surgical characteristics were extracted. The publications were arranged chronologically and presented as a historical timeline (Figure 1).

To illustrate the clinical application of the proposed treatment algorithm, we present a representative case from our institution together with the corresponding reconstructive strategy. A 52-year-old male patient presented with a traumatic partial amputation of the superior third of the auricle following a dog bite injury. The defect involved the upper and middle portions of the helix, the scaphoid fossa, and the superior crus of the antihelix, corresponding to a Grade III injury according to the classification of Laskin and Donohue. Owing to the absence of suitable recipient vessels for microsurgical replantation, a two-stage auricular reconstruction was performed using a modified pocket technique. This approach was selected to maximize tissue preservation and optimize both functional and aesthetic outcomes in the setting of a non-replantable auricular amputation.

## 3. Results

The chronological development of auricular reconstruction techniques following traumatic amputation was analyzed and documented over a period of more than 120 years, from 1898 to 2018 (Table 1). To facilitate comparison and improve interpretability, the identified milestones were categorized according to their underlying reconstructive principles and summarized in a chronological timeline (Figure 1).

The earliest documented approach was reported by Brown in 1898, who described the use of a composite graft for auricular reconstruction [11]. More than five decades later, Sexton introduced the pocket method in 1955 [9], which subsequently became one of the most influential techniques for the management of non-replantable auricular amputations. Several modifications of this principle were developed in the following decades. Converse described the placement of the amputated auricle within an abdominal pocket in 1958 [24,25], a concept later adopted by Musgrave and Garrett in 1967 [12]. Gerow proposed storage within a supraclavicular pocket, whereas Conroy utilized a cervical pocket. The principal distinction among these techniques was the anatomical location selected for temporary implantation of the amputated auricular segment.

However, distant pocket techniques were associated with several drawbacks, including the considerable distance from the defect site, prolonged immobilization, distortion of the auricular framework, tissue contraction, partial cartilage resorption, and suboptimal aesthetic outcomes. In contrast, Sexton and later Mladick et al. advocated the use of a postauricular or mastoid pocket located adjacent to the defect [9,10]. This approach allowed preservation of the native auricular contour while minimizing tissue deformation. Following retrieval from the pocket, Mladick’s technique relied on spontaneous re-epithelialization of the exposed cartilage framework, supplemented by split-thickness or full-thickness skin grafting when necessary [10]. Sexton, in contrast, elevated the replanted segment together with the overlying skin and covered the resulting donor-site defect with either split-thickness or full-thickness skin grafts [9].

Further refinements were introduced by Mladick and Carraway in 1973, resulting in the modified pocket method [13]. Their technique incorporated transcartilaginous mattress sutures to secure the skin more firmly to the replanted auricular cartilage framework, thereby promoting closer tissue contact and enhancing the rate of successful re-epithelialization. In parallel, Spira et al. described an alternative variation involving distant tissue banking of the amputated auricular segment in 1974 [26].

Alternative strategies based on composite grafting were introduced by Baudet et al. in 1972, who reported the use of multiple cartilage fenestrations to facilitate vascular ingrowth and improve graft survival [14]. This concept was subsequently expanded by Arfai et al. in 1974 [26].

The 1970s also marked the emergence of autologous cartilage reconstruction techniques. Brent et al. first described auricular reconstruction using autologous costal cartilage in 1974 [15], thereby establishing the foundation for modern total auricular reconstruction. This concept was later refined and popularized by Nagata et al. in 1993, whose modifications significantly improved aesthetic outcomes and reproducibility [16].

A major milestone in the evolution of auricular reconstruction was the introduction of microsurgical replantation. Miller et al. reported the first successful microsurgical replantation of an amputated auricle in 1976 [17]. Subsequent advances were described by Nahai et al. in 1978 [18] and Pennington et al. in 1980 [19], establishing microsurgical revascularization as a viable reconstructive option for selected patients with suitable vascular anatomy.

During the late 1980s, several authors proposed local flap-based reconstruction techniques as alternatives to replantation. These approaches were reported by Ariyan et al. in 1986 [20], Anous et al. in 1988 [21], and Jenkins et al. in 1989 [22]. Local tissue transfer provided additional reconstructive options for cases in which replantation was not feasible or had failed.

The most recent paradigm shift was represented by the introduction of tissue-engineering approaches. In 2018, Zhou et al. described bioengineered strategies for auricular reconstruction, highlighting the potential of regenerative medicine, biomaterial-based scaffolds, and three-dimensional bioprinting technologies for the development of patient-specific reconstructive solutions [23].

The complete chronological overview of these milestones is presented in Table 1 and illustrated in Figure 1.

## 4. Discussion

**Figure 2:**
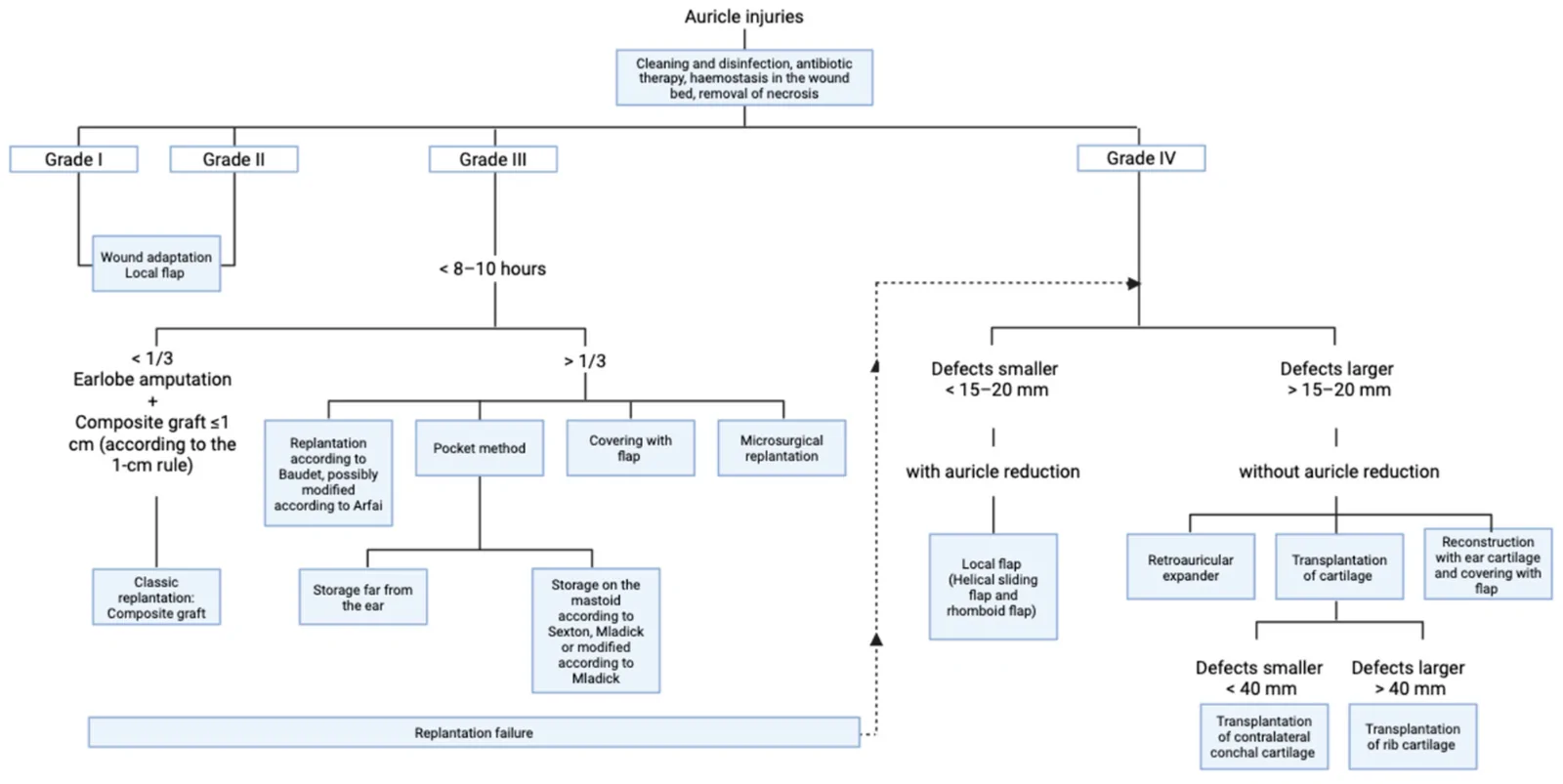
Proposal for a therapeutic algorithm for managing auricular injuries based on the modified classification system of Laskin and Donohue (1958) [7]. Created in BioRender. https://app.biorender.com/illustrations/.

As previously outlined, auricular injuries are most associated with traffic injuries, bite traumas or other complications [1]. As a general principle, partial or complete loss of the auricle leads to three-layer lacerations, which can be reattached using various surgical techniques. Below, we present an algorithm that summarizes the general treatment strategies based on the commonly used classification (Figure 2). It is important to note that this represents a selection and recommendation for clarity and overview but does not encompass all possible reconstructive methods.

For general preoperative management, we recommend thorough wound cleansing and disinfection with Granudacyn^®^ as early as possible, along with the initiation of antibiotic therapy, particularly if the wound has been present for more than 24 h. Debris should be meticulously removed, and the wound irrigated with Betaisodonal solution. Adequate hemostasis within the wound bed is essential. Previous studies have indicated that infections are infrequent when treatment is administered promptly. Earley and Bardsley identified anaerobic bacteria as the primary pathogens responsible for infection after human bites, thereby advocating antibiotic therapy with penicillin or ampicillin [27].

In the case of a grade 1 injury to the pinna involving skin flaps with minimal cartilage damage, we recommend precise wound adaptation using 6-0 or 7-0 monofilament sutures, applied cutaneously. Smaller defects can be covered with surrounding tissue flaps. For grade 2 auricular avulsions, where a tear exists but a nourishing skin bridge remains, we advise attempting readaptation as the initial approach. This involves suturing the skin on the posterior aspect using 6-0 monofilament sutures with an atraumatic, fine needle. The cartilage is then repaired using 4-0 or 5-0 nonabsorbable monofilament sutures, followed by closure of the anterior skin with 6-0 monofilament sutures. In cases where defects persist, they are covered with adjacent skin flaps [8]. Grade 3 injuries involving fresh avulsion without segment loss present a particular challenge and should be managed through reattachment of the detached segment. In cases of complete auricular loss, replantation of the fully severed tissue sections is possible. However, due to the narrow, vascularized base, a high rate of tissue loss is anticipated as a result of trophic disturbances, depending on the size of the replant [11,28,29]. In cases of complete amputation, the detached auricle should be transported in a sterile compress within a replantation bag whenever feasible. The bag should be cooled to approximately 4 °C by placing it in a mixture of ice cubes and water. Direct contact between the tissue and the ice water must be avoided to prevent frostbite.

According to the proposed treatment algorithm, Grade III injuries involving less than one-third of the auricle, including isolated earlobe amputations, may be managed by conventional (classic) replantation using a multilayer reconstructive approach [8]. In this technique, the amputated auricular segment is meticulously reattached through layered reconstruction of the skin and cartilaginous framework. Depending on the extent and configuration of the defect, either skin–cartilage composite grafts (two-layer composite grafts) or skin–cartilage–skin composite grafts (three-layer composite grafts) may be employed.

This approach is generally recommended for partial auricular amputations involving less than one-third of the auricular framework, where diffusion-mediated vascularization is considered sufficient to support graft survival. Nevertheless, the outcomes of classic replantation have been inconsistent, particularly in larger defects, due to the very narrow, nourishing base of the wound. In response to these limitations, Baudet et al. criticized the relatively poor success rates associated with conventional composite graft reattachment and introduced the concept of a two-layer composite graft with fenestrated cartilage to facilitate neovascularization and improve graft viability [14]. Between 1972 and 1993, a total of 13 cases utilizing this technique were reported in the literature. Among these, complete graft survival was achieved in 38% of cases, whereas total graft loss occurred in 23% [8,30,31]. These findings underscore the inherent limitations of non-vascularized composite grafting in the reconstruction of larger auricular defects. Overall, graft failure rates of approximately 20% have been reported for conventional replantation techniques, although favorable outcomes may be achieved with appropriate patient selection and meticulous surgical technique [32,33]. The “1-cm rule” by Brown and Cannon states that the maximum diffusion distance from the vascularized wound bed to the most distant portion of a free auricular composite graft should not exceed approximately 1 cm [11]. Larger grafts are at increased risk of ischemia and partial or complete necrosis due to insufficient vascularization. Therefore, defects exceeding 1 cm should be managed according to the same reconstructive principles as injuries involving more than one-third of the auricle [29,32,33].

Regardless of the specific composite graft method employed, strict postoperative immobilization of the replanted segment for 6–7 days remains a critical determinant of success. Immobilization protects the fragile neovascular connections established during the early postoperative period, thereby facilitating graft integration and tissue survival [32,33].

For auricular amputations involving more than one-third of the auricular framework, conventional composite graft reattachment is associated with substantially reduced survival rates, necessitating the use of alternative reconstructive strategies. Consequently, considerable efforts over the past several decades have focused on improving graft viability through techniques that promote neovascularization prior to definitive reconstruction. Among these, various forms of the pocket technique have emerged as the most widely adopted approaches. Several anatomical sites have been proposed for temporary implantation of the amputated auricular segment, including abdominal pockets [12,34], supraclavicular pockets [26], and cervical pockets [35]. However, these distant implantation techniques frequently resulted in separation of the cartilaginous framework from the overlying soft tissues, marked distortion of the auricular architecture, partial resorption of cartilage, and unsatisfactory aesthetic outcomes, often rendering the preserved tissue unsuitable for definitive auricular reconstruction [8,36]. Nevertheless, distant implantation may still be indicated in patients with extensive traumatic defects of the periauricular head and neck region, where local tissue conditions preclude implantation adjacent to the defect. To overcome these limitations, later modifications favored implantation in close proximity to the defect site. Placement within the mastoid region, as described by Sexton and subsequently refined by Mladick and colleagues [9,10,13], provided improved preservation of the native auricular contour and facilitated subsequent reconstruction. Alternative approaches included coverage of the amputated auricular segment with vascularized local flaps, such as the myocutaneous platysma flap [20] or the temporoparietal fascial flap [37].

Notably, immediate auricular reconstruction using local flaps alone has generally yielded suboptimal outcomes in several clinical series [1,30,38]. Direct reconstruction is therefore discouraged because of the high risk of postoperative contraction, distortion of the auricular framework, and consumption of valuable local skin reserves, which may compromise secondary reconstructive options in the event of graft failure.

**Figure 3:**
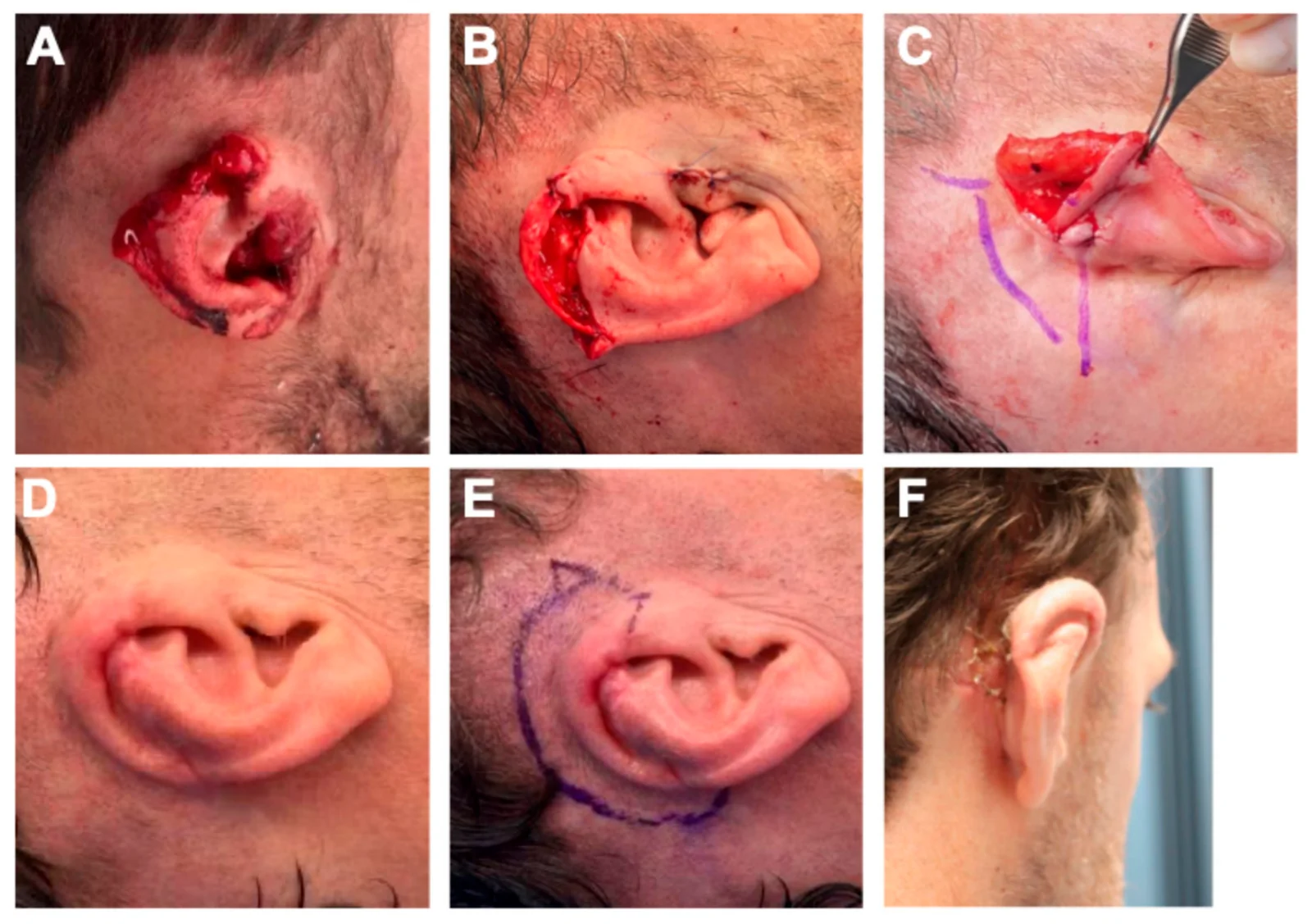
A 52-year-old male presented with a traumatic amputation of the cranial third of the auricle due to a dog bite. (**A**) Traumatic injury involving the upper and middle third of the auricle, characterized by partial avulsion of the pinna without complete amputation and classified as a Grade III injury. (**B**) Following debridement, the auricular cartilage framework was repositioned to ensure anatomical alignment. The amputated cartilage-bearing segment was secured to the remaining auricular cartilage using figure-of-eight sutures, restoring the continuity of the helical rim. (**C**) Preoperative marking of the subcutaneous mastoid pocket to bury the reattached cartilage. (**D**) After 8 weeks, well-vascularized and fully integrated cartilage framework within the mastoid pocket, demonstrating preservation of key auricular landmarks, including the helix, antihelix, and scapha. (**E**) Design of an advancement galeal flap based on the temporoparietal fascia for reconstruction of the posterior auricular surface, with cranial marking of a Burrow’s triangle. (**F**) Clinical appearance three weeks postoperatively. The replanted auricular segment shows successful integration, with viable, well-perfused overlying skin and complete healing of the full-thickness skin graft. The helix, superior crus of the antihelix, and scaphoid fossa are clearly defined, and normal auricular projection has been preserved. The angle between the mastoid plane and auricular plane is ≤30°, and the distance from the mastoid plane to the upper edge of the helix is ≤20 mm.

The term “pocket technique” was first introduced by Mladick et al. in 1971 [10]. In their original description, the amputated auricular segment underwent bilateral dermabrasion before being implanted in an anatomically correct position beneath the postauricular mastoid skin. In contrast, Sexton’s earlier technique involved complete removal of the anterior and posterior skin envelopes while preserving the cartilaginous framework, which was subsequently implanted beneath the retroauricular mastoid skin [9]. Figure 3 illustrates this operative principle, in which the skin and subcutaneous tissue are excised entirely, leaving only the auricular cartilage framework intact, consistent with Sexton’s original method. A key distinction between the techniques of Sexton and Mladick lies in the management of the skin envelope. Whereas Sexton removed the skin completely, Mladick et al. employed dermabrasion, thereby removing only the epidermis while preserving the underlying dermis and its vascular plexus [10]. In cases with insufficient local soft-tissue coverage, additional local flaps were used to ensure adequate burial of the cartilage framework. Following successful incorporation, the skin lining of the pocket formed the new anterior auricular surface. During the second stage, the reconstructed auricle was elevated after 8 weeks, and the posterior aspect was covered using a thick split-thickness or full-thickness skin graft harvested from donor sites such as the upper arm, thigh, groin, or gluteal region [24,25].

This concept was subsequently refined by Mladick and Carraway through a modification of the pocket technique, in which transcartilaginous mattress sutures were used to secure the skin more firmly to the replanted auricular framework [13]. The rationale behind this modification was to enhance nutritional support of the graft by improving contact between the skin envelope and the underlying cartilage, thereby reducing tissue contraction and minimizing postoperative shrinkage. In the three cases reported by Mladick and Carraway, elevation of the auricle was performed after only 14 days of implantation, with favorable outcomes observed in all patients [13]. Only one case demonstrated a small area of incomplete epithelialization.

In the present case, the skin was similarly secured to the cartilaginous framework using mattress sutures according to the technique described by Mladick et al. [13]. However, unlike Mladick’s dermabrasion-based approach, complete excision of the skin and subcutaneous tissue was performed in accordance with the original method described by Sexton [9]. This strategy combined the advantages of Sexton’s complete cartilage banking technique with the improved skin-to-cartilage fixation proposed by Mladick, resulting in a modified pocket technique for auricular reconstruction that has not previously been described in this form [31].

A critical technical aspect of the procedure is the fixation of the cartilaginous framework. Inadequate attachment of the amputated cartilage to the residual auricular stump may result in posterior folding or displacement of the framework during implantation within the pocket. To avoid this complication, the pocket should be prepared generously, allowing tension-free placement of the cartilage framework while maintaining its anatomical orientation. In selected cases, additional fixation of the auricular remnant to the adjacent mastoid periosteum or planum mastoideum may be required to ensure stable positioning and preserve the three-dimensional contour of the reconstructed auricle [8,39].

If suitable vascular structures can be identified within the amputated segment, microsurgical replantation is generally the preferred treatment. Successful replantation requires at least one suitable artery, whereas venous anastomosis is not always mandatory if adequate venous drainage can be achieved by alternative means. However, the absence of venous repair is associated with an increased risk of venous congestion, partial tissue necrosis, and a greater need for postoperative blood transfusions. Miller et al. [17] and Nahai et al. [18] were among the first to pioneer this approach, achieving successful replantation of a partially amputated auricle. Subsequently, Pennington et al. reported the first successful complete ear replantation in 1980 [19]. In a 30-year follow-up of the first successful microsurgical ear replantation, Pennington et al. demonstrated durable long-term viability and satisfactory aesthetic outcomes [40,41]. In a subsequent systematic review of microsurgical ear replantation, Momeni et al. reported that 63.3% of cases were managed with combined arterial and venous anastomoses, whereas 31.7% underwent arterial anastomosis alone with venous drainage through alternative means [42]. The review by Steffen et al. further demonstrated that microsurgical replantation was associated with the highest success rates in cases of complete auricular amputation, whereas the pocket technique yielded the most favorable outcomes for partial auricular amputations [38].

In cases of auricular amputation where replantation is not feasible, particularly in grade 4 injuries involving segmental loss, alternative reconstructive strategies must be considered. Small full-thickness defects of the helix or antihelix (<15–20 mm or <15% of auricular height) may be successfully managed with primary closure or local flap reconstruction [43,44]. However, closure of larger defects using these techniques may result in noticeable auricular distortion; therefore, the reconstructive approach should be tailored to the defect size, residual auricular anatomy, and anticipated aesthetic outcome. Available options include auricular reduction procedures utilizing helical sliding flaps or rhomboid flaps, as originally described by Dufourmentel [8]. Another option is replacing the lost cartilage with a rib cartilage graft, typically from the 6th, 7th, or 8th rib, sculpted to be 2–3 mm smaller than the reconstructed auricle [43,44,45]. Alrawi et al. recommended contralateral conchal cartilage grafts for defects up to 4 cm and autologous rib cartilage graft for defects more than 4 cm [46]. The choice of donor site should also be guided by the patient’s age, as well as the pliability and structural characteristics of the costal cartilage. In cases with insufficient skin, such as after burns, a 35 mL expander is implanted postauricularly and gradually expanded for about 8 weeks, after which the skin is used for reconstruction [44,47]. Tissue engineering offers new possibilities for treating partial auricular defects and auricular aplasia [48]. In animal models, preformed transplants made from chondrocytes and biocompatible materials have shown biomechanical properties similar to human nasal septal cartilage within 12 weeks [48,49,50]. A significant milestone in the field of tissue engineering was the development of the Vacanti mouse, which demonstrated the successful transplantation of chondrocytes using a polymer-based cellular scaffold to generate tissue-engineered cartilage that replicated the morphology of a human ear [51]. The first clinical application of in vitro-generated, patient-specific ear-shaped cartilage for auricular reconstruction was reported in 2018 in 5 cases [23]. This development marked an important step toward personalized auricular reconstruction and highlighted the potential of tissue engineering and three-dimensional bioprinting to generate patient-specific cartilage constructs. Despite these promising advances, the clinical translation of tissue-engineered and 3D-bioprinted auricular constructs remains limited by several key challenges. These include insufficient vascularization, inadequate long-term mechanical and structural stability, difficulties in achieving reproducible chondrogenic differentiation, and a lack of robust long-term clinical safety and outcome data [52,53,54].

## 5. Conclusions

Auricular reconstruction following traumatic amputation has evolved from composite grafting and pocket techniques to microsurgical replantation and emerging tissue-engineering approaches. Based on this historical evolution, we propose an individualized treatment algorithm guided by tissue loss and residual anatomy. Limited injuries can generally be managed by primary closure or local flaps, whereas larger partial or complete amputations require consideration of composite grafting, pocket techniques, flap reconstruction, or microsurgical replantation when suitable vessels are available. Complete tissue loss requires secondary reconstruction tailored to defect size and available tissue. Tissue engineering and 3D bioprinting may further expand patient-specific reconstructive options in the future.

## Figures and Tables

**Table 1:** Chronological evolution of auricular reconstruction techniques.

Study	Reattachment/Replantation Technique	Date	Reference
Brown	Composite graft	1898	[11]
Sexton	Pocket method	1955	[9]
Converse	Pocket method	1958	[24,25]
Musgrave and Garrett	Pocket method storage far from the ear	1967	[12]
Mladick et al.	Pocket method	1971	[10]
Baudet et al.	Composite graft with fenestrated cartilage	1972	[14]
Mladick and Carraway	Modified Pocket Method	1973	[13]
Spira et al.	Pocket method storage far from the ear	1974	[26]
Arfai et al.	Composite graft with fenestrated cartilage	1974	[26]
Brent et al.	Autologous rib cartilage	1974	[15]
Miller et al.	Microsurgical replantation	1976	[17]
Nahai et al.	Microsurgical replantation	1978	[18]
Pennington et al.	Microsurgical replantation	1980	[19]
Ariyan et al.	Local flap	1986	[20]
Anous et al.	Local flap	1988	[21]
Jenkins et al.	Local flap	1989	[22]
Nagata et al.	Autologous rib cartilage	1993	[16]
Zhou et al.	Tissue engineering	2018	[23]

## Data Availability

No new data were created or analyzed in this study. Data sharing is not applicable to this article.

## Supplementary Materials

The following supporting information can be downloaded at: https://www.mdpi.com/article/10.3390/jcm15186942/s1. File S1: PubMed search strategy.
