# Supplementary material for: Auricular Reconstruction Following Traumatic Amputation: A Historical Review and Contemporary Treatment Algorithm
Source: J Clin Med. 2026 Sep 8;15(18):6942. doi: 10.3390/jcm15186942 (PMC13607691; doi:10.3390/jcm15186942)
Supplement: Supplementary file 1 [file jcm-15-06942-s001.zip › jcm-4512586-supplementary.pdf]

Supplementary Material File S1:

("Ear, External"[Mesh] OR "Ear Auricle"[Mesh] OR auricle*[Title/Abstract] OR auricular[Title/Abstract] OR "external ear"[Title/Abstract] OR ear[Title/Abstract]) AND ("Amputation, Traumatic"[Mesh] OR "Replantation"[Mesh] OR "Amputation Stumps"[Mesh] OR amputation*[Title/Abstract] OR avulsion*[Title/Abstract] OR amputat*[Title/Abstract] OR replant*[Title/Abstract] OR reattach*[Title/Abstract] OR "reattachment"[Title/Abstract]) AND ("composite graft"[Title/Abstract] OR "composite grafts"[Title/Abstract] OR "pocket method"[Title/Abstract] OR "pocket principle"[Title/Abstract] OR "modified pocket"[Title/Abstract] OR microsurg*[Title/Abstract] OR "microvascular anastomosis"[Title/Abstract] OR "local flap"[Title/Abstract] OR "rib cartilage"[Title/Abstract] OR "autologous cartilage"[Title/Abstract] OR "tissue engineering"[Title/Abstract]).
